# Predictive value of PAR and PNI for the acute complicated course of pediatric acute hematogenous osteomyelitis

**DOI:** 10.1016/j.jped.2024.04.002

**Published:** 2024-04-25

**Authors:** Chaochen Zhao, Zhiye Guan, Qizhi Jiang, Wangqiang Wu, Xiaodong Wang

**Affiliations:** a Children's Hospital of Soochow University, Department of Orthopaedics, Suzhou, Jiangsu Province, China; b Shanghai Jiao Tong University, School of Medicine, Shanghai Children's Hospital, Department of Orthopaedics, Shanghai, China

**Keywords:** Acute hematogenous osteomyelitis, Platelet to albumin ratio, Prognostic nutritional index, Complication

## Abstract

**Objective:**

Platelet to albumin ratio (PAR) and prognostic nutritional index (PNI) are potential indicators for evaluating nutritional and inflammatory status. This study aimed to examine the relationship between PAR and PNI and the acute complicated course of acute hematogenous osteomyelitis (AHO).

**Methods:**

AHO patients were divided into the simple course group and the acute complicated course group. The patient's gender, age, site of infection, body temperature, laboratory results, and pathogen culture results were collected and compared. Multivariate logistic regression analysis was used to determine the independent risk factors of the acute complicated course group. The receiver operating characteristic curve was applied to determine the optimal cut-off value.

**Results:**

In total, 101 AHO patients with a median age of 7.58 years were included. There were 63 cases (62.4 %) in the simple course group and 38 cases (37.6 %) in the complicated course group. Binary logistic regression analysis revealed that PAR and PNI were independent risk factors for predicting the acute complicated course of AHO (*p* = 0.004 and *p* < 0.001, respectively). Receiver operating characteristic curve analysis demonstrated that the combination of PAR and PNI had an area under the curve of 0.777 (95 % CI: 0.680–0.873, *p* < 0.001) with a cut-off value of 0.51.

**Conclusions:**

The incidence of acute complicated courses was significantly higher in patients with high PAR and low PNI. A combined factor greater than 0.51, derived from PAR and PNI measurements within 24 h of admission, may be useful for predicting AHO patients who are likely to develop severe disease.

## Introduction

Acute hematogenous osteomyelitis (AHO) is a relatively prevalent infectious disease among children. Clinical symptoms typically include fever, pain, swelling of the affected area, impaired mobility, and difficulty in weight-bearing. The main route of infection is bacterial blood-borne transmission, and prompt antibiotic treatment is crucial to prevent sepsis, chronic osteomyelitis, septic arthritis, and other complications. The incidence of AHO has been reported to range from 2 to 40 cases per 100,000 individuals, with a slightly higher incidence in boys compared to girls.^1^, ^2^, ^3^, ^4^, ^5^ Most patients respond well to empiric intravenous antibiotics.^6^ However, some children require several surgeries and prolonged hospitalizations and even experience short-term recurrence.^7^ Hence, it is essential to identify children who need complex treatment courses at an early stage. Magnetic resonance imaging (MRI) is a reliable method for accurate assessment of soft tissue and joints, with high sensitivity and specificity in diagnosing AHO. However, improvement in imaging findings may not always keep pace with clinical progress, and MRI is not suitable for predicting complex disease courses.^8^ In terms of laboratory findings, although several severity of illness scoring frameworks (e.g., Copley score, A-score, C-score, Gouveia score)^9^, ^10^, ^11^ have confirmed the feasibility of serial C-reactive protein (CRP) tests in evaluating the complex course of AHO, it is essential to consider there are other factors that can elevate CRP levels. Therefore, investigation of other simple and effective biomarkers that can facilitate early and rapid screening of children who may develop a complicated course of AHO is needed. Such a tool may enable the implementation of individualized management strategies, shorten hospital stays, and help guide decisions on oral antibiotic therapy and surgical interventions.

Platelet to albumin ratio (PAR) and prognostic nutritional index (PNI) are easily obtained in clinical settings and have been recognized as novel prognostic indicators for several infectious diseases. Baba et al.^12^ discovered that an increased preoperative PAR is a risk factor for infection following heart transplantation. Similarly, Huang et al.^13^ reported that PAR is positively correlated with the disease activity of axial spondyloarthritis. Additionally, Li et al.^14^ found that PNI is inversely and independently associated with the presence and severity of neonatal sepsis. However, there is still a lack of studies investigating the use of PAR and PNI in predicting the complicated course of AHO.

Therefore, this study aimed to evaluate the clinical value of PAR and PNI obtained within hours of admission in predicting the acute complicated course of pediatric AHO.

## Methods

This study was approved by the Medical Ethics Committee of the Children's Hospital of Soochow University (approval number 2021KS024). The requirement for informed patient consent was waived.

Patients diagnosed with AHO who were admitted for inpatient treatment at the hospital between January 2016 and January 2023 were retrospectively recruited for this study. All participant data were obtained retrospectively from their medical records.

### Study design, setting, and study subjects

The inclusion criteria were as follows: (1) confirmed diagnosis of AHO; and (2) presentation of symptoms for 14 days or less prior to hospital admission, coupled with the absence of antibiotic treatment prior to conducting blood tests.^15^ The exclusion criteria were as follows: (1) patients with underlying conditions, such as hematologic diseases, rheumatic diseases, heart diseases, malignancies, or chronic diseases; (2) postoperative infections; and (3) patients with incomplete medical information. Diagnostic criteria for AHO included the presence of causative organisms in blood culture, pus culture, synovial fluid culture, and tissue culture, as the widely accepted standard for diagnosis. When culture results were negative, an indirect diagnosis was made in case of clinical symptoms of AHO, positive laboratory and imaging findings, and response to antibiotics within 48 h.^16^^,^^17^

### Standard treatment protocol

Patients with AHO, presenting symptoms for up to 14 days before admission, were started on intravenous empirical antibiotics. The continuation of this regimen awaited bacteriological and antibiotic sensitivity results for appropriate adjustments. Should the infection rapidly progress, or symptoms not improve noticeably within 2–4 days post-antimicrobial therapy initiation, surgical options were evaluated, particularly with MRI findings of intraosseous abscesses or subperiosteal abscesses larger than 2 cm. Patients responding well to medical treatment, evidenced by improved gastrointestinal function, reduction in inflammatory signs, and two consecutive CRP levels below 8 mg/L, were switched from intravenous to oral antibiotics. Those requiring surgery or with abscess formation had prolonged intravenous therapy, with oral transition tailored to their recovery. It's crucial to note the variability in treatment decisions across clinical teams.^18^

Children with AHO were divided into the acute complicated course group and the simple course group. The acute complicated course was defined based on the need for ≥ 2 surgical procedures, hospitalization for more than 21 days, or recurrence within six weeks of intravenous antibiotic therapy.^9^, ^10^, ^11^^,^^19^ This definition was established based on guidelines proposed by Alhinai et al.^11^ and Gouveia et al.,^10^ particularly incorporating a hospitalization duration of over 21 days as a criterion for an acute complicated course to accommodate local treatment practices. Distinct from the patterns observed in Western countries, the epidemiological characteristics, virulence, and antibiotic resistance profiles of prevalent pathogens in AHO, such as *Staphylococcus aureus*, vary significantly in Asia.^20^^,^^21^ Consequently, local treatment protocols are more conservative, often requiring that patients exhibit two consecutive CRP levels below 8 mg/L before transitioning to oral antibiotics. Adopting the criterion of more than 21 days of hospitalization as a marker for complex disease courses is intended to precisely reflect the local therapeutic approaches and challenges, as evidenced by Gouveia et al.’s research.

Patient data collected included: gender, age, site of infection, body temperature, culture results, erythrocyte sedimentation rate (ESR), white blood cell count (WBC), CRP, neutrophil count, lymphocyte count, monocyte count, platelet count and serum albumin, within 24 h of admission. The authors then calculated PAR and PNI. PAR was defined as platelet to serum albumin ratio, and PNI was defined as albumin (g/L) + 5 *lymphocyte count (*10^9^/L). Fever on admission was defined as a body temperature greater than 38.5 °C in children upon hospitalization.

### Statistical analysis

Data were analyzed using IBM SPSS Statistics Version 26.0 (IBM Corporation, Armonk, NY). Continuous variables with a normal distribution are presented as mean ± standard deviation. Homogeneity of variance was assessed using a student-*t*-test, and a corrected-*t*-test was used for variance heterogeneity. Median and IQR (25th, 75th percentiles) was recorded for data with a non-normal distribution, and the Wilcoxon rank-sum test was applied. Categorical variables were expressed by presenting the frequency and proportion in each category. Categorical variables were analyzed using Chi-square analysis. Variables exhibiting statistical significance in univariate analysis were included in multivariate analysis to identify independent risk factors. The logistic model equation was then applied to construct a combined indicator. Receiver operating characteristic curve (ROC) analysis was used to establish the area under the curve (AUC) for independent risk factors and their combination. Differences were considered significant when *p* < 0.05.

## Results

**Figure 1:**
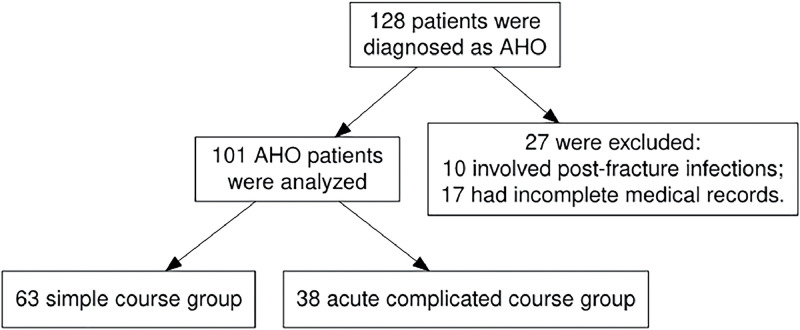
The flowchart of the retrospective study.

In total, 128 patients were diagnosed with AHO. Of these, 27 were excluded from the study for the following reasons: 10 cases involved post-fracture infections, and 17 had incomplete medical records. The latter group included 15 patients who were transferred to the present department after receiving initial treatment in other hospitals or departments, and 2 who discontinued their treatment and self-discharged. As a result, 101 patients were ultimately enrolled in the study. The flowchart is shown in Figure 1.

In total, 101 patients with AHO were included in this study, comprising 58 males and 43 females. The site of infection was as follows: 28 cases (27.7 %) in the tibia, 24 cases (23.7 %) in the femur, 14 cases (13.8 %) in the humerus, 10 cases (9.9 %) in the calcaneus, 5 cases (5 %) in the fibula, 5 cases (5 %) in the pelvis, 5 cases (5 %) in the fingers, 4 cases (4 %) in the toes, 2 cases (2 %) in the radius, 2 cases (2 %) in the patella, 1 case (1 %) in the clavicle, and 1 case (1 %) in the spine. Pathogen cultures were conducted for 85 pediatric patients, with 77 undergoing bacterial blood cultures. The positivity rate for blood cultures stood at 14.3 % (11/77), with *Staphylococcus aureus* being identified in all 11 instances. In cases where cultures pus, synovial fluid, or tissue were obtained through surgical intervention in 39 instances, the results were positive in 76.9 % (30/39) of the cases, with *Staphylococcus aureus* emerging as the predominant pathogen (27 cases, 90 %). Additionally, one case of *Salmonella enterica, Klebsiella pneumoniae*, and *Acinetobacter baumannii* was identified (each constituting 3.3 % of positive cultures). Overall, pathogens were detected in 35 cases (41.2 %), with *Staphylococcus aureus* accounting for 32 instances. Among these, 23 cases were diagnosed with methicillin-sensitive *Staphylococcus aureus* (MSSA), all of which exhibited sensitivity to the initially administered *β*-lactam antibiotics (cefuroxime or ceftriaxone). Conversely, 9 cases were identified as methicillin-resistant *Staphylococcus aureus* (MRSA), for which treatment regimens were adjusted to vancomycin following antibiotic susceptibility testing. Furthermore, the antibiotic treatment strategies for the cases caused by *Salmonella enterica, Klebsiella pneumoniae*, and *Acinetobacter baumannii* were also tailored based on the respective antibiotic sensitivity profiles. Moreover, among all cases, there were three instances of multifocal infections and no cases of disseminated disease (such as pneumonia, septic pulmonary embolism, deep vein thrombosis, or endocarditis) were identified. The aim of this study was to investigate the predictive value of clinical indicators within the first 24 h of admission for the development of an acute complicated course in pediatric AHO. Given that disseminated or multifocal infection cases typically manifest in the later stages of the disease and were relatively rare in this sample, these conditions were not included within the scope of this study's observations.

The study included 101 patients, divided into the simple course group (63 cases) and the acute complicated course group (38 cases). In the simple course group, there were 36 males and 27 females, with a median age of 7.92 years. Among them, 21 (33.3 %) cases had fever on admission. Similarly, 22 males and 16 females were in the acute complicated course group, with a median age of 7.29 years. Among them, 15 cases (39.5 %) had fever on admission. There were no significant differences in age, gender, and presence of fever between the 2 groups (*P* > 0.05). Univariate analysis revealed significant differences in baseline measurements between the acute complicated course group and the simple course group: CRP, ESR, and PAR levels were significantly higher (*p* < 0.05), and PNI levels were significantly lower (*p* < 0.05) in the complicated course group, whereas differences in WBC levels were not statistically significant (*p* > 0.05) (Table 1).

**Table 1:** Univariate analysis of general information and laboratory indicators of AHO patients enrolled in the study (*n* = 101).

Variables	Simple course (*n* = 63)	Acute complicated course(*n* = 38)	*χ²/ t / Z*	*P*
Gender, *n* (%)			0.005	0.941
Male,	36 (57.1)	22 (57.9)		
Female	27 (42.9)	16 (42.1)		
Age (years), Median (IQR)	7.92 (2.83-11.25)	7.29 (3.29-10.73)	−0.025	0.980
Fever on admission, *n* (%)	21 (33.3)	15 (39.5)	0.390	0.533
WBC (×10^9^/L), Median (IQR)	11.13 (8.27-13.22)	11.67 (7.63-13.79)	−0.060	0.952
CRP (mg/L), Median (IQR)	31.32 (3.00-74.05)	60.42 (21.29-104.12)	−2.117	0.034
ESR (mm/L), Median (IQR)	25 (13-47)	52 (34.5-68.5)	−3.099	0.002
PAR, Median (IQR)	6.95 (5.60-8.65)	8.45 (6.39-10.45)	−2.236	0.025
PNI, Median (IQR)	58.75 (53.80-67.10)	53.78 (46.70-60.05)	−3.449	0.001

WBC indicates white blood cell count; CRP, C-reactive protein; ESR, Erythrocyte sedimentation rate; PAR, Platelet to albumin ratio; PNI, prognostic nutritional index.

The authors applied multifactorial logistic regression analysis, using the conditional backward method for single-factor indicators with statistical significance in one factor, revealing that PAR and PNI independently correlated to the acute complicated course of AHO (*p* = 0.004 and < 0.001). While CRP and ESR were not independent risk factors (Table 2). On admission, children with higher PAR and lower PNI were more likely to develop the acute complicated course. The formula for the combined indicator was calculated from the regression coefficients in Table 2 (*L* = 4.605 + 0.206 * PAR - 0.120 * PNI).

**Table 2:** Binary logistic regression analysis backwards stepwise (conditional) for acute complicated course.

Variables	*Β*	Standard Error	*χ*²	p	Odds Ratio	95 % CI for Odds Ratio Lower Upper	
PAR	0.206	0.072	8.216	0.004	1.229	1.067	1.415
PNI	−0.120	0.032	13.942	<0.001	0.887	0.833	0.945
Intercept	4.605	1.676	7.552	0.006	100.002	——	

PAR indicates Platelet to albumin ratio; PNI, prognostic nutritional index.

The results of ROC curve analysis for PAR, PNI, PAR+PNI, CRP, and ESR to predict the acute complicated course are shown in Table 3; the AUC were 0.633, 0.706, 0.777, 0.626, and 0.685, respectively. Among them, ESR had the highest sensitivity but the lowest specificity, while CRP had the lowest sensitivity but higher specificity. PAR and PNI jointly yielded the highest AUC, with an optimal cut-off value of 0.51. Considering a Youden index of 0.5, a sensitivity of 57.9 %, a specificity of 92.1 %, a positive predictive value of 81.6 %, a negative predictive value of 78.4 %, and an accuracy of 79.2 % were obtained. The combined indicator demonstrated better predictive value for the acute complicated course of AHO than the univariate analysis.

**Table 3:** Comparison of ROC curve analysis for predicting acute complicated course.

Variables	AUC	Cutoff value	Sensitivity	Specificity	PPV	NPV	Youden index	Accuracy
PAR	0.633	7.76	0.632	0.65	0.521	0.745	0.283	0.643
PNI	0.706	55.19	0.632	0.683	0.568	0.755	0.315	0.663
PAR+PNI	0.777	0.51	0.579	0.921	0.816	0.784	0.5	0.792
CRP	0.626	84.43	0.395	0.825	0.576	0.693	0.220	0.663
ESR	0.685	31.5	0.789	0.444	0.461	0.777	0.345	0.574

PPV indicates positive predictive value; NPV, negative predictive value; CRP, C-reactive protein; ESR, Erythrocyte sedimentation rate; PAR, Platelet to albumin ratio; PNI, prognostic nutritional index.

## Discussion

In the present retrospective study, the authors aimed to investigate the predictive value of PAR and PNI in predicting the acute complicated course in AHO patients. The results showed that using PAR and PNI indicators within 24 h of admission may aid in the early detection of children with AHO who will experience a complicated disease course. Although patients with AHO are empirically treated with intravenous antibiotics, there is considerable variation in disease severity among individuals, necessitating a personalized management approach to either adopt conservative or aggressive treatment strategies. In addition, nutritional support, guided by a dietitian and involving oral or parenteral supplementation of proteins and energy, plays a pivotal role in the treatment of pediatric AHO. While CRP and ESR are instrumental in assessing the disease progression in AHO, reliance on these biomarkers requires serial measurements within 2–4 days for accurate evaluation. Delays in assessment could potentially impact the timely performance of high-resolution imaging examinations, lead to unnecessary invasive management, and affect the implementation of adjunct nutritional support therapies. Therefore, exploring novel serum biomarkers that enable early and rapid screening of children at risk of acute complicated course of AHO may be useful in facilitating precision therapy.

Pathogen infections disrupt the balance between neutrophils, lymphocytes, and platelets, escalating inflammatory responses, which reflects the immune system's state.^22^^,^^23^ Nutritional status plays a crucial role in maintaining health and preventing infections. Serum albumin regulates inflammation, maintains vascular integrity, and affects drug distribution and elimination. It is considered one of the most direct indicators of nutritional status.^24^ PAR and PNI, calculated based on serum albumin, platelet count, and lymphocyte count, more comprehensively reflect the nutritional and immune status. Moreover, PAR and PNI are less likely to be influenced by dynamic physiological conditions. Zhai et al.^25^ discovered that PAR can predict the progression of persistent acute kidney injury in ICU patients. They also found that a PAR value of 7.2 can be used as a cut-off point for early risk stratification. Liu et al.^26^ reported a positive correlation between PAR level and disease activity in patients with rheumatoid arthritis. Additionally, a multicenter study by Wei et al.^27^ revealed a significant association between PNI and the prognosis and mortality of COVID-19 patients. As emerging biomarkers, PAR and PNI are gradually gaining attention in clinical applications due to their operational simplicity and cost-effectiveness, highlighting their potential utility.

The characteristics of this cohort, including the median age of patients, gender distribution, and causative organisms, were consistent with previous reports of AHO.^28^ Furthermore, the present findings demonstrated that CRP and ESR, as traditional indicators of inflammation, had an AUC of 0.626 and 0.685, respectively. Similarly, Martin et al.^29^ reported that CRP and ESR can predict the acute complicated course of AHO. CRP and ESR can serve as crucial indicators for early identification of the complicated course of pediatric AHO. However, when employing backward elimination for variable selection, these markers were not included in this study due to their high correlation with other factors and the necessity for serial measurements within 2–4 days for reliable assessment.^9^^,^^11^ This study focuses on indicators that effectively predict outcomes within 24 h, thus CRP and ESR were excluded from the analysis. The present results identified a correlation between on-admission PAR and PNI and the likelihood of experiencing a complicated disease course. Binary logistic regression analyses illuminated that PAR and PNI are independent predictors for the acute complicated course of AHO. The combined indicator yielded an AUC of 0.777, higher than individual indicators. These findings suggest that the combination of PNI and PAR may be used to help predict the acute complicated course of AHO. While this combination demonstrates a high specificity in the predictive model, it is important to acknowledge the limitation presented by its lower sensitivity. Consequently, further external validation of these predictive tools is imperative to confirm their clinical utility.

PAR and PNI levels can also be used to guide adequate supplementation of calories and protein. Cha et al.^30^ reported that In critically ill patients with sepsis, the mortality decreased as the amount of protein or energy supply increased. As a consequence, when the combined indicator value is greater than 0.51, the authors should pay attention to nutrition management. This study represents a pioneering effort to assess the efficacy of novel serum biomarkers, measured within 24 h of admission, in predicting the acute complicated course of AHO, highlighting the innovative aspect of this research. Despite this, it is crucial to recognize that the present findings, at this juncture, cannot supplant existing predictive tools; they serve merely as a supplementary aid and are still in a nascent stage, necessitating further validation. The future plans involve integrating these novel, albumin-related biomarkers, specifically PNI and PAR, with current scoring systems to refine the predictive model. Notably, the present study's team has developed a new predictive model that synergizes these novel biomarkers with radiological findings and clinically relevant variables. This model has been preliminarily compared with the A-score and Gouveia scoring systems in a local cohort, demonstrating better predictive capability. However, this model requires further optimization and rigorous validation. The results of this study are pending publication.

### Limitations

This study has several limitations. First, the sample size was small, and the standardized definition of the complicated course was missing. There was also a selection bias as the authors grouped patients based on treatment experience in the studied region. Second, due to insufficient follow-up length, the long-term prognostic values of PAR and PNI were not analyzed. Third, all AHO patients were Chinese, which limits the generalizability of the results. Therefore, further prospective studies are needed to confirm the present results.

## Conclusions

In conclusion, elevated PAR and reduced PNI may aid in identifying patients with AHO who were at risk of developing acute complicated courses. Although this combined assessment exhibits a high degree of specificity, its lower sensitivity suggests it may be more appropriately utilized as an adjunctive tool to enhance the performance of other diagnostic instruments, rather than as a standalone predictive measure. The combined indicator of PAR and PNI may be considered in the development of future risk assessment tools to improve their predictive accuracy.

## Conflicts of interest

The authors declare no conflicts of interest.
